# King Edward's Hospital Fund

**Published:** 1910-03-12

**Authors:** 


The Hospital

A Journal of

The Medical Sciences and Hospital Administration.

New Series. No. 160, Vol. VI. [No. 1232, Vol. XLVII.] SATURDAY, MARCH 12, 1910.

### KING EDWARD'S HOSPITAL FUND.

The thirteenth annual report of this Fund, which will be found in another column of the present issue, is a remarkable document in many ways. We may say in passing that we believe 13 is the King's favourite number, and certainly the thirteenth report of his Fund nobly justifies the confidence His Majesty expressed in the statement which he issued as Prince of Wales on February 5, 1897. At that time there were not wanting many critics who prophesied failure, or were altogether opposed to the Fund, chiefly because it was maintained that it must interfere with the voluntary revenue of individual hospitals. This fear has proved to be without foundation, and is finally laid to rest by the Fund's gifts of £150,000 in 1909. In fact, so far from diminishing the voluntary revenue of the hospitals, the institution of the King's Fund and the League of Mercy, by widening the area of givers, has tended to increase the gifts which are each year received from voluntary sources by individual hospitals.

The King's Fund has deservedly won the confidence of the hospitals and the public, for it has been exceptionally well and tactfully managed from the commencement. This is confirmed by the facts that since the inauguration of the Fund the amount spent on administration has been less than 1¼ per cent. of the total amount received, and that the cost of management shows practically no increase during the last two years. When we examine the accounts and balance-sheets, the phenomenal success attained is at once apparent. The capital and general funds now amount in round numbers to £1,720,000, and yielded an income in 1909 of £68,502, which is in round figures equal to 4 per cent. The balance of income was received from legacies, £121,401; League of Mercy, £19,000; annual subscriptions, £23,615; donations, £12,145. The King's original letter proposed to raise the revenue from three sources, with a yield of £50,000 a year from each. Investments, thanks mainly to the personal service and work of the Prince of Wales, the President, have exceeded this estimate annually; subscriptions and donations, which have never been actively worked for fear of competing with individual hospitals, are £14,000 below the estimate; and the smaller contributors have yet to supply a further £31,000 a year. The League of Mercy paid over £21,000 to the Fund and certain provincial hospitals in 1909, so that it has still much to do if it is to raise £50,000 a year from small subscriptions of 1s. and upwards. Its members have done splendid service in the past, and we have little doubt that when there is less unemployment and better business the contributions from this source will steadily grow. It is just and it is also popular to speak well of the King's Fund and its work, but we should be better pleased with these encomiums if the names of more than a relatively few people appeared in the subscription and donation lists of the Fund every year. If every subscriber to the Fund at present would get a friend to adopt the same course, the £50,000 a year from this source would be readily forthcoming within the next twelve months.

The general work of the Fund has been of infinite value to the hospitals, and has tended to benefit them directly both financially, economically, and administratively, and as indirectly by raising the standard of efficiency of all the voluntary hospitals throughout the country. The annual statistical reports issued by the Fund have proved of great value and have led to a considerable saving in the cost of commodities and in other directions. The work of the Visitors, and the annual and careful inspections they make of all the voluntary hospitals in London every year have been fruitful for good. The Distribution Committee has not only won for itself a high position by the justice and care with which it has distributed the money given to the hospitals each year, but by a careful examination of all schemes for the extension or reconstruction of hospitals it has helped to prevent wasteful expenditure upon buildings, and to provide that each extension shall be so planned as best to fulfil the purposes in view. A further and valuable work of the Distribution Committee has been to keep always in mind in relation to each scheme of reconstruction what bearing it has on the general question of hospital accommodation throughout London, and on the prospect of funds being forthcoming for its maintenance after erection. The Convalescent Homes Committee has done good work, and the negotiations with consumption sanatoria in the country with a view to place beds at the disposal of hospitals in London for suitable cases have a paramount importance.

We are glad to notice that Mr. John Burns, the President of the Local Government Board, has appointed Messrs. Deloitte, Plender, Griffiths and Co. to audit the accounts of the Fund, for they have rendered good service in the past, and one of the founders of the firm, Mr. John G. Griffiths, has proved a most helpful member of the General Council and its committees. The Fund has it in contemplation to appoint a committee to deal with the question of out-patients during the present year. This is a thorny subject which requires drastic treatment, and it is of the utmost importance that the members of any such committee shall be selected with great care, if permanent good is to result. We could wish that the negotiations for the amalgamation of the throat, nose, and ear hospitals would show some signs of progress. As matters stand at present these institutions, like Mahommed's coffin, are suspended in mid-air, so far as any direct grants from the King's Fund are concerned. May we suggest, for the consideration of the committees of these institutions, that it would be well for them to meet privately in conference, to consider the whole question on its merits, to prepare a careful report, and then to submit it, as promptly as possible, by a deputation to the King's Hospital Fund.

